# Identifying Geogenic and Anthropogenic Aluminum Pollution on Different Spatial Distributions and Removal of Natural Waters and Soil in Çanakkale, Turkey

**DOI:** 10.1021/acsomega.2c07707

**Published:** 2023-02-22

**Authors:** Sezin Hızlı, Aybike Gül Karaoğlu, Ayşegül Yağmur Gören, Mehmet Kobya

**Affiliations:** † Department of Environmental Engineering, Gebze Technical University, 41400 Gebze, Turkey; ‡ Department of Environmental Engineering, Izmir Institute of Technology, 35430 Urla, İzmir, Turkey; § Department of Environmental Engineering, Kyrgyz-Turkish Manas University, Bishkek 720044, Kyrgyzstan

## Abstract

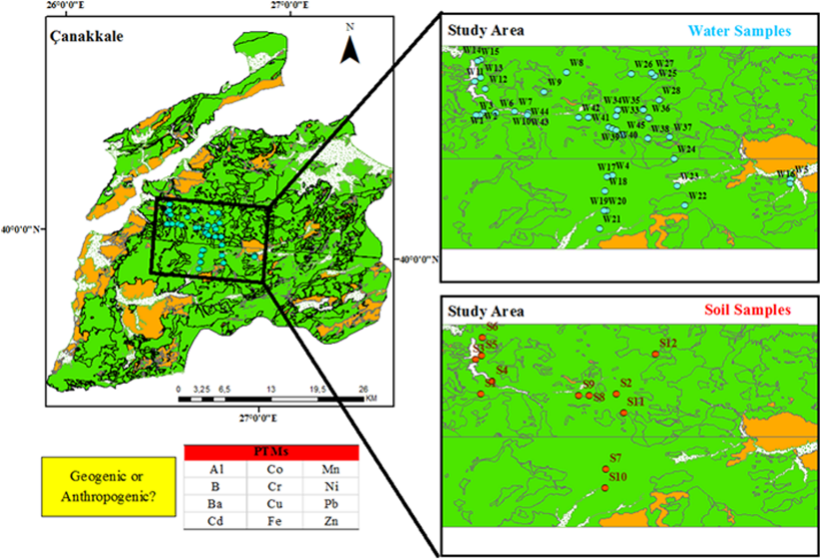

The Çanakkale–Kirazlı region (Turkey) is enriched with minerals, especially aluminum (Al), which dangerously get transported into aquatic media due to several mining and geological activities in recent years. In this study, Al and other potentially toxic metals (PTMs) including B, Ba, Cd, Co, Cr, Cu, Fe, Mn, Ni, Pb, Si, and Zn, in both water and soil samples, were measured for quality determination. Selected metals were also analyzed by the enrichment factor (EF), the geoaccumulation index (*I*_geo_), the contamination factor (CF), and the pollution load index (PLI) to evaluate both water and soil pollution geogenically or anthropogenically. Also, the metals were clustered to support the pollution source with Pearson’s correlation, principal component analysis (PCA), and hierarchical cluster analysis (HCA). Forty-five natural water samples and 12 soil samples were collected spatially. To perform pollution assessment, two fundamental treatment processes to remove Al pollution from the sample including the highest Al concentration (38.38 mg/L) in water were applied: (1) precipitation with pH adjustment and (2) removal with ion exchange. The pH values of water samples were changed in the range of 3–9 to test the dissolution of Al. The results demonstrated that the study area was mostly under the influence of geogenic aluminum pollution.

## 1 Introduction

Aluminum (Al) is the third most abundant element in the earth’s crust, comprising about 8.8% by weight (88 g/kg), and occurs naturally in combination with oxides and silicate minerals. Clays and other secondary minerals range from 45% A1 for boehmite to 3% A1 for glauconite. Of the sedimentary rocks, shales generally have the highest content of A1 (7.8–8.2%), followed by sandstones (2.5–4.2%) and carbonates (0.4–1.3%).^1^ Al enters environmental media naturally through the weathering of rocks and minerals. Anthropogenic releases are in the form of air emissions, industrial effluents, and solid wastes. High aluminum concentration in an aquifer due to low pH is caused by geogenic (natural) and anthropogenic factors. The latter are mostly acid mine or rock drainages processes,^2−4^ redundant alum usage, or lack of treatment of domestic and industrial wastes,^5,6^ but the former occurs naturally with interaction of water–rock or geothermal fluid–geological formation (rock), and are generally the main reason for the huge amount of Al transferred from the soil into natural water sources.^7−9^ The amount of aluminum in natural waters varies from 0.0001 to 1 mg/L, and in acidic waters (pH < 5), the concentration of aluminum may even exceed 100 mg/L. Aluminum compounds show low solubility in the pH range of 6–8; therefore, in surface water and groundwater, the concentrations of aluminum are in the range of 0.060–0.30 mg/L.^10^

Interaction between rock and water, including Al solubility and speciation, is supported by the acidic pH values and affects the quality of drinking water as well as the environment it reaches.^11,12^ Al species tend to be soluble and form ligands with inorganic and organic matters at pH below 5 in natural waters by acid rain or acid mine tailings or at pH above 8.^1^ Mobility and transport of Al ions into the water change with the generated sulfate concentration by oxidation of sulfureous soil minerals, the composition of the geological materials, the coordination chemistry, and the flow of water in acidic environments, which is influenced by especially troublesome phenomena such as acid mine drainage (AMD).^13^ Mining activities result in many metals getting mobilized and reacting with water and the atmosphere from the surrounding rock, causing exposition and reaction of the pyrite mineral, which form a solid metal hydroxide complexation and decrease the pH by sulfuric acid production, thereby increasing the toxic metal concentrations in aquatic media.^3,14^ Because of the obtained high solubilization capacity, the concentration of Al found in these waters can reach up to 90 mg/L.^15^ Al is becoming a major contributor to environmental problems, not only causing diseases, illnesses, and disorders (i.e., Alzheimer’s disease, gastrointestinal illnesses, dementia, kidney or liver function disorders^16−18^) but also entering the food chain owing to its bioaccumulative and nonbiodegradable properties;^19,20^ hence, it has to be removed from wastewaters in related facilities properly. The United States Environmental Protection Agency (USEPA) and the World Health Organization have maximum allowable aluminum concentrations of 0.05–0.2 and 0.20 mg/L in drinking water, respectively.^13,21^ Potentially toxic metals (PTMs), especially heavy metals, are currently removed using many water treatment methods such as coagulation–flocculation,^22^ electrocoagulation,^23,24^ ion exchange,^25,26^ adsorption,^27−29^ and membrane processes.^30,31^

To date, several studies have been conducted on heavy metal contamination in the soil, sediment, and water in Turkey.^32−35^ For instance, the heavy metal contamination of groundwater resources in the Bafra Plain was evaluated considering geostatistical and ordinary kriging approaches.^36^ The authors reported that the Al, As, Fe, and Mn concentrations were above the levels permissible for drinking waters, with a considerably high heavy metal pollution index of 21.97%. In a separate study, an assessment of the health risk and ecotoxicological parameters was conducted considering potentially toxic elements (Al, As, Cd, Cu, Cr, Co, Fe, Mn, Ni, Pb, U, and Zn) in sediments for some rivers of Giresun, especially located in hazelnut production areas.^37^ Al and Fe were the dominant elements in sediments, with high concentrations compared with other metals, and Al concentrations were in the range of 27 869–45 060 mg/kg. On the other hand, the contaminant factor (CF) of Al with 0.5 revealed that the Al in all sediment samples causes a low level of contamination (CF < 1). In addition, the health risk assessment results showed that the hazard index (HI) values of elements were ranked in the following order: Fe > Co > As > Al > Pb > Cr > U > Mn > Cu > Ni > Cd > Zn. Overall, there was no significant noncarcinogenic toxicity of selected elements as HI values were less than 1. The contaminant levels of heavy metals in a subtropical river basin system of Giresun were also studied by Ustaoğlu and Aydın.^38^ It was reported that the contamination level of Al (267 μg/L) in the river was considerably above the WHO permissible levels (200 μg/L). Moreover, the Nemerow pollution index, which presents individual information taking standard values into consideration about the contamination degree of pollutants as well as focuses on key pollutants, was determined for all heavy metals, and the values were in the range of 0–1.43. These results revealed that only Al metal had a significant impact on heavy metal load in all river samples. The principal source of metals in rivers may thus be lithological, with no significant anthropogenic heavy metal pollution. In the Melet River (Ordu, Turkey), which is surrounded by agricultural fields, heavy metal concentrations most probably originating from agricultural residues, mining activities, and household residues were determined in water and sediments.^39^ The heavy metal concentrations were reported in the following order: Fe > Al > Mn > As > Zn > Cu > Ni > Cr > Cd = Pb = C and Fe > Al > Mn > Zn > Cu > Pb > Cr > As > Co > Ni > Cd in water and sediment media, respectively. Similar to the previous studies performed in the Giresun rivers, Al and Fe were found to be the most dominant metals in the Ordu river sediment and water samples. Furthermore, the spatial-temporal pollution indices and distribution of heavy metals in Ordu at the Turnasuyu stream sediment were assessed systematically by considering seasonal samples from various sites.^40^ As expected, average concentrations of 15 080 and 6416 mg/kg were observed for Fe and Al elements, respectively. Furthermore, the calculated mean geoaccumulation index values of −4.23 for Al and −2.23 for Fe revealed that the sediment samples were unpolluted with Al and Fe and there was no environmental risk. In most of these reviewed studies, specific research on aluminum contaminations in soil, sediment, and water environments is insufficient. Furthermore, studies on environmental risk assessment considering Al are limited, according to our humble opinion. Therefore, there is a crucial need for a comprehensive study on the assessment of environmental risks of Al pollution as well as monitoring of Al contamination in water and soil media.

Although geogenic Al pollution has been seen in different regions of Turkey, this study attempts to determine potentially toxic metal (PTM) pollution in both natural waters and soils and assess the source of the pollution using the enrichment factor (EF), geoaccumulation index (*I*_geo_), contamination factor (CF), and pollution load index (PLI) on the samples collected from Kirazli, Çanakkale. Additionally, correlation of the metals with the source was owing to multivariate analyses (Pearson’s correlation, principal component analysis (PCA), and hierarchical cluster analysis (HCA)). Finally, two economically feasible removal methods were applied to remove Al: pH adjustment and ion exchange.

## 2 Materials and Methods

### 2.1 Study Area

**Figure 1:**
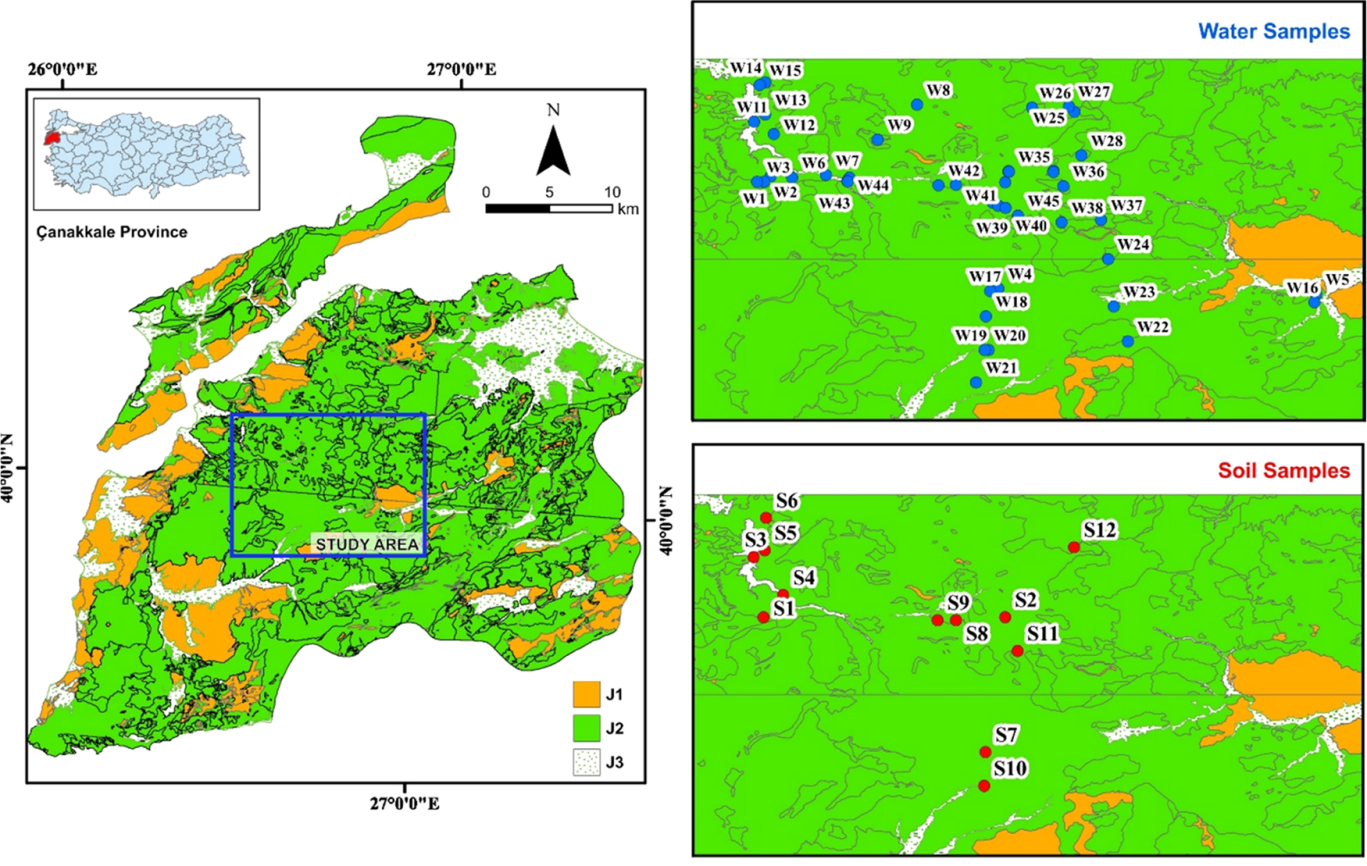
Study area of the Kirazlı region.

The study area of 1115.3 km^2^ is located in Northwestern Turkey within the Çanakkale province (Figure 1). Kirazlı village is located about 40 km southeast of the city center and around the Biga Peninsula, which is an active tectonic region. Mountainous topography features are seen in the region. Kirazlı Mountain is the most important hill in the region, 811 m above the sea level and covered with forests, which provides the main means of livelihood for the local people. In this peninsula, alternating reddish-yellow-white-colored volcanic and sedimentary rock formations are commonly seen.^41^ The former formations are altered Neogene-age sedimentary covered with sand, silt, and clay,^16^ and both formations are covered by quaternary alluvium, including sand and gravel grains. In the rock structures of the region, lead (Pb)–zinc (Zn)–copper (Cu) and gold (Au) metal deposits and industrial minerals such as clay (Al_2_O_3_·2SiO_2_·2H_2_O), coal, and kaolinite (Al_2_Si_2_O_5_(OH)_4_) have been identified.^42^

In Çanakkale, Biga and some nearby towns (Yenice, Can, and Lapseki) are known for having a total of 204 metallic mineral deposits, and the most important ones are Cu, Pb, Zn, antimony (Sb), and gold (Au) reserves. Volcanic units at Kirazlı belong to the Miocene age, which host alternating zones and precious metal mineralization and contain feldspar, mafic minerals, and some quartz. The enrichment of metals is Al + K in the argillic and Mg + Ca + Fe in the propylitic alteration types. Moreover, two Au mineral deposit reserve places are found—Kartal Dag and Maden Dag—and deposits of Fe and Mn also have found been as small mass reserves. Environmental changes (causing geogenic interaction between soil and water) affect the enrichment and leaching of metals; for example, Ca, Mg, and Fe were leached during argillic alteration, whereas strong Na leaching is evident in all alteration types.^43^

The hydrogeology of the Kirazlı region generally comprises volcanic units. Most of the springs in the study area are between the silicified zone and the argillic zone. Several springs surface from volcanic soils such as tuff and agglomerate in the Biga Peninsula. These springs have flow rates between 0.01 and 3 L/s. In the region Çanakkale and Koca streams discharge into the Atikhisar Reservoir, which serves the water supply system of Çanakkale city.^41^ Generally, the main alluvial aquifers in the region serve as the main water resources.^41^ As seen in Figure 1, the study area has three types of geological structures. J1, J2, and J3 represent, respectively, high mineral soil, low mineral soil, and alluvial soil. While J1 includes evaporite mineral sedimentary rocks such as gypsum and carbonates with high solubility only in acidic waters, travertine, caliche, limestone, marble, and calcschist formations, J2 consists of aluminum silicate-containing soils, conglomerates, sandstone, and silica-predominant formations.^44^ X and Y in Figure 1 indicate the geologic coordinates, whereas W and S indicate water and rock samples, respectively. The peninsula is in the Mediterranean and Black Sea transition zone, affecting climate characteristics, with summers being hot and dry and winters being cold and rainy. Maximum precipitation is observed during the winter, whereas the least precipitation is observed during summer.^42^

### 2.2 Sample Collection and Analysis

Sampling locations were determined with the help of GPS coordinates (GARMIN GPS eTrex 30x) surrounding Kirazlı village. Water and soil samples were collected during the dry season (on September 6–7, 2019). Water samples, including surface water (*n* = 3, nos.: W11 (dam water), W2, and W32 (stream water)) and groundwater (*n* = 42, nos. 1–45, apart from W11, W2, and W32), were collected in polyethylene bottles (500 mL), with the following sampling and analytical procedure carried out using the Standard Methods for the Examination of Water and Wastewater.^45^ Electrical conductivity (EC), total dissolved solid (TDS), dissolved oxygen (DO), and pH were measured on-site. Additionally, total alkalinity, sulfate ion (SO_4_^2–^), and metal analysis were conducted at the laboratory of the Environmental Engineering Department of Gebze Technical University. The metals investigated within the scope of this study were selected by taking into account the metals and metalloids in the soil and water samples as a result of the preliminary analysis by an inductively coupled plasma-optical emission spectrophotometer (ICP-OES, Optima 7000 DV, PerkinElmer). As a result of the preanalysis, metals such as As, Cr, Hg, and V were not detected in the samples; therefore, these metals were not considered in the study. Consequently, total concentrations of 15 metals (Al, B, Ba, Ca, Cd, Co, Cr, Cu, Fe, Mg, Mn, Ni, Pb, Si, and Zn) were analyzed by ICP-OES.

Each of the surface soil samples (∼500 g) was collected from close to the springs at 0–10 cm (upper soil layer) soil samples (*n* = 12 S1–S12) and collected into polyethylene bags. All samples were transferred to the laboratory and stored at 4 °C. Before being ground to <100 μm with a mortar, the soil samples were dried at 105 ± 2 °C for 48 h. Then, 0.25 g of sample was exposed to 2 mL of HNO_3_, 2 mL of HF, 1 mL of HCl, and 1 mL of H_2_O_2_ in Teflon vessels for 24 min and digested in a model Milestone Ethos 1600 advanced microwave digestion apparatus. Then, each digestate was diluted to 50 mL with ultrapure water, and the resulting solution was analyzed for the 15 metals with the water samples by ICP-OES. All reagents used were of analytical grade. X-ray diffraction (XRD, Bruker D-8 Advance) was applied for mineralogical identifications on randomly collected soil samples. The identification was also supported by scanning electron microscopy (SEM, Philips XL 30S-FEG, The Netherlands) equipped with energy-dispersive X-ray spectroscopy (EDS, AMETEK Inc.).

### 2.3 Data Management and Statistical Analysis

Before performing multivariate data analysis, the min, max, mean, and standard deviation (SD) of the data set were calculated to determine the coefficient (metal and physicochemical parameters) variation of sampling locations. The statistical analysis was performed by SPSS (IBM, version 21.0) using the Pearson correlation coefficient matrix, principal component analysis (PCA), and hierarchical cluster analysis (HCA) to show the correlation between elements and physicochemical parameters to assess pollution origin.

### 2.4 Assessment of PTM Contamination with Pollution Indices for the Soil Matrix

#### 2.4.1 Enrichment Factor (EF)

EF was computed to assess the type and degree of PTM pollution in the studied soils.^46^ It helps determine whether the pollution source is anthropogenic or geogenic.^47^ It is calculated using eq 1, where Me is the metal concentration in the soil and Ref is the reference metal. In this case, Fe was used as the reference metal instead of aluminum.^48^ This metal can be a reference or background material because it is also an abundant metal on the earth, it has no outlier, and it was normally distributed, as obtained by the normality test and Box–Whisker plots.^49,50^
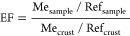
1EF results were classified as 0–1, 1–3, 3–5, 5–10, 10–25, 25–50, and >50, indicating no enrichment, minor enrichment, moderate enrichment, moderately severe enrichment, severe enrichment, very severe enrichment, and extremely severe enrichment, respectively.^50,51^

#### 2.4.2 Geoaccumulation Index (*I*_geo_)

The metal pollution index is a measure of soil quality by evaluating single substances. It was introduced by Müller to evaluate the measured metal concentrations by comparing preindustrial levels in sediments.^52^ It is widely used in defining river sediment quality in studies, but this index is also preferred to express metal pollution in soils.^53,54^
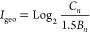
2In eq 2, *C_n_* is the current metal (*n*) concentration in the soil and *B*_n_ is the geochemical background value (BGV) of the metal in the sample. The factor 1.5 is the coefficient for the background matrix coming from geogenic variations. *I*_geo_ was categorized into six classes:^52^ <0: unpolluted; 0–1: unpolluted to moderately polluted; 1–2: moderately polluted; 2–3: moderately to strongly polluted; 3–4: strongly polluted; 4–5: strongly to extremely polluted; and >5: extremely polluted.

#### 2.4.3 Contamination Factor (CF) and Pollution Load Index (PLI)

CF is used for determining toxic metal pollution in soils.^55^ It is calculated as shown in eq 3, where *C*_Me_ is the metal concentration in the soil and *C*_n_ is the geochemical background concentration of the metal. This factor is defined using four classifications: CF < 1: low contamination; 1 ≤ CF < 3: moderate contamination; 3 ≤ CF < 6: considerable contamination; and CF > 6: very high contamination.
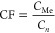
3
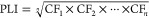
4PLI is calculated from CF and can help define the pollution site quality based on the concentration of each metal in the soil.^56^ In Equation 4, *n* is the number of metals possibly toxic to the site. When PLI < 1, it means that the background and raw data are similar and there is no pollution, and when PLI > 1, it indicates pollution by the metals analyzed.

## 3 Results and Discussion

The results of the study are submitted in four parts. The first two parts are about determination of PTMs and the physicochemical parameters in water samples. The third part is determination of the soil quality, and the fourth part presents the efficiency of Al removal by precipitation and ion-exchange methods. Additionally, the second and third parts evaluate the pollution source of PTMs.

### 3.1 Concentrations of PTMs in Natural Water Samples

The physicochemical parameters (pH, EC, TDS, DO, alkalinity, and sulfate) and metal concentrations measured from analysis of water samples are shown in Table 1, with drinking water standards of WHO,^57^ Turkish Drinking Water Quality Standards (TDWQS)^58^ with A1–A3 classes, and Turkish regulation on waters for human consumption (WHC).^59^ In TDWQS, classes A1, A2, and A3 represent, respectively, water that becomes high-quality potable raw water after simple physical treatment and disinfection; slightly polluted water that becomes potable after physical treatment, chemical treatment, and disinfection; and poor-quality water that becomes potable after physical treatment, chemical treatment, advanced treatment, and disinfection. WHC explains water is hygienically and technically suitable for drinking by humans. The pH of the water samples ranged between 3.33 and 9.92, with an average of 6.03; the maximum pH was at W6 and the minimum was at W41.

**Table 1:** Measured Physicochemical Parameters and Metal Concentrations in Water Samples of Kirazlı and Nearby Villages

					TWQS (quality classification)				
parameter	unit	min	max	mean	A1	A2	A3	WHC	WHO
pH		3.330	9.920	6.029	6.5–9.5	6.5–9.5	6.5–9.5	6.5–9.5	ND
T	°C	14.00	20.40	18.20	ND^a^	ND	ND	ND	ND
EC	μS/cm	87.00	1493	593.27	2500	ND	25000	ND	ND
TDS	mg/L	44.80	733.0	294.22	ND	ND	ND	ND	ND
DO	mg/L	4.210	10.17	8.311	ND	ND	ND	ND	ND
ALK	mg/L	0.00	465.0	151.07	ND	ND	ND	ND	ND
SO_4_^2–^	mg/L	16.68	567.4	130.04	250	ND	1250	250	ND
Al	mg/L	0.027	38.38	3.217	0.2	0.5	2	0.2	0.2
B	mg/L	0.000	0.596	0.058	1	1.25	5	1	2.4
Ba	mg/L	0.011	0.254	0.048	2	ND	20	ND	1.3
Ca	mg/L	3.723	190.7	59.16	ND	ND	ND	ND	ND
Cd	mg/L	0.002	0.114	0.010	0.005	0.015	0.050	0.005	0.003
Co	mg/L	0.001	0.032	0.011	0.8	ND	2.6	ND	ND
Cr	mg/L	0.003	0.955	0.100	0.05	0.5	1	0.05	0.05
Cu	mg/L	0.001	0.263	0.022	2	5	20	2	2
Fe	mg/L	0.014	14.78	1.247	0.2	1	2	0.2	ND
Mg	mg/L	0.670	57.00	15.03	ND	ND	ND	ND	ND
Mn	mg/L	0.006	2.131	0.223	0.05	0.1	0.25	0.05	ND
Ni	mg/L	0.002	0.050	0.022	0.02	0.03	0.2	0.02	0.07
Pb	mg/L	0.002	0.634	0.094	0.01	0.05	0.1	0.01	0.01
Si	mg/L	3.394	60.96	18.25	ND	ND	ND	ND	ND
Zn	mg/L	0.001	0.320	0.065	3	6	12	ND	ND

a ND, no data.

It is recommended by TDWQS (class A1) and the WHO that the pH should be within 6.5–9.5, but according to the mean value of the pH, this sampling site was found to be acidic in nature. Acidic waters dissolve chemical constituents, affect the transport of toxic elements in water, and might harm aquatic organisms^11^ or human beings. The water temperature differed between 14 and 20.4 °C, which affects the availability of inorganic constituents (PTMs) and the growth of microorganisms.^57^ There is no information about EC to compare the measured values in the water, and it was on average 593.3 μS/cm, with the maximum at the W22 and the minimum at the W4 sampling site. TDS was mostly in classes I and II range with a mean concentration of approximately 294.2 mg/L. DO levels ranged from 4.21 to 10.17 mg/L; the lowest DO level was from well number W1 close to Çiftlikdere. Sulfate ions fluctuated from 16.68 to 567.40 mg/L, with the average value being 130.4 mg/L. The high values of the ions might be due to the pollution caused by acidic mining drainage and soil weathering.^60^ Ba, Cd, Cr, Cu, and Pb metals were within the toxic limits of TWQS, WHC, and WHO. Some metals such as Co, Fe, Mg, Mn, Si, and Zn have no limit of concentration defined by WHO; however, these metals should be monitored in drinking waters since these metals act as indicators for pollution before water treatment becomes obligatory. On the other hand, B may be in the suitable range for drinking, but it is an indication of anthropogenic pollution. Because in previous studies^61−63^ B was not determined in soil or rock analysis, while Al, Mg, Mn, Fe, and Si were found, it can be said that the metal presence comes from human activities.

### 3.2 Assessment of PTMs and Physicochemical Parameters in Waters

#### 3.2.1 Pearson Correlation Matrix

Pearson’s correlation was studied to investigate the association between PTMs and physicochemical parameters (Table 2). Kirazlı is enriched in many mineral deposits, especially gold; therefore, mining activities have been going on for years and evidence of geogenic interaction can be seen in both groundwater and surface water. Due to silicified, propylitic, and argillic alterations and especially aluminum silicate-dominant formations, Si, Al, Ca, and Mg were found in the groundwater and surface water samples.

**Table 2:** Pearson Correlation for Water Samples of Kirazlı^a^

	EC	TDS	DO	SO_4_^2–^	Fe	Mn	B	Al	Ca	Mg	Si	Co	Ni	Zn
EC	1.000													
TDS	**1.000**	1.000												
DO	–0.261	–0.251	1.000											
SO4	**0.673**	**0.668**	–0.143	1.000										
Fe	0.060	0.064	–0.231	0.143	1.000									
Mn	***0.297***	***0.298***	0.037	**0.534**	**0.509**	1.000								
B	**0.456**	**0.450**	**–0.384**	***0.373***	0.146	–0.010	1.000							
Al	–0.013	–0.015	0.147	***0.345***	0.053	**0.428**	–0.153	1.000						
Ca	**0.856**	**0.855**	–0.291	**0.479**	0.003	0.207	***0.313***	–0.183	1.000					
Mg	**0.469**	**0.478**	0.003	0.214	–0.029	0.141	0.166	–0.190	**0.572**	1.000				
Si	0.200	0.206	0.091	**0.412**	***0.324***	**0.546**	–0.104	**0.508**	0.112	–0.058	1.000			
Co	0.083	0.084	0.200	0.096	–0.166	–0.119	0.208	–0.067	0.066	–0.124	–0.029	1.000		
Ni	–0.160	–0.158	0.061	–0.023	0.190	0.083	–0.270	0.005	–0.228	***–0.304***	0.140	***0.353***	1.000	
Zn	0.071	0.064	–0.044	***0.369***	0.088	**0.587**	0.021	**0.475**	–0.067	–0.158	0.145	–0.062	0.003	1.000

a Bold values indicate that the correlation is significant at the 0.01 level (two-tailed); bold and italic values indicate that the present correlation is significant at the 0.05 level (two-tailed).

The results indicate that they all mostly have positive correlations between each other. While TDS represents dissolved ions and is mostly related to the aquifer rock geochemistry, it is strongly possible to have a direct correlation with EC (1.000), SO_4_^2–^ (0.668), and naturally found metals in waters, such as Ca (0.855) and Mg (0.478). Both TDS and EC are closely related to the number of ions present in the water,^64^ and it is supported by the Pearson correlation. Al has significant positive correlations with Si (0.508), Zn (0.475), Mn (0.428), and SO_4_^2–^ (0.345). SO_4_^2–^ has correlation nearly with all variables such as Mn (0.534), Ca (0.479), Si (0.412), Zn (0.369), B (0.373), and Al (0.345) besides DO, Fe, Mg, Co, and Ni. The presence of SO_4_^2–^ in groundwater and surface water samples is geogenically due to pyrite oxidation, which occurs mostly from the soil weathering process by AMD, and this ion can be used as an indicator.^3,14,60^

#### 3.2.2 PCA and HCA

**Figure 2:**
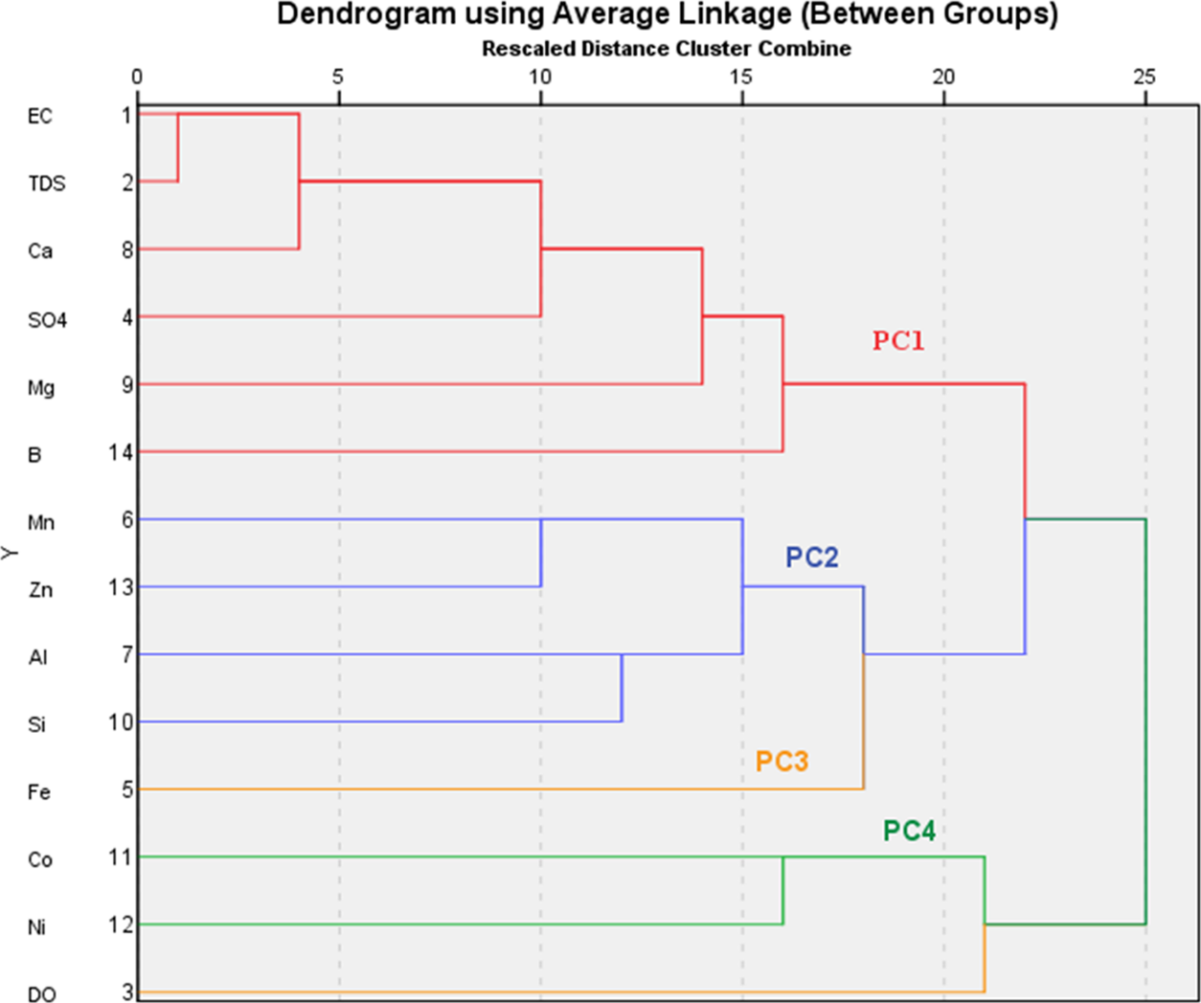
Dendrogram of HCA for PTMs and other variables (Pearson correlation, between-group linkage method).

PCA is a method of factor analysis, and it was applied to concentrations of PTMs and physiochemical parameters of water samples for presenting how spatial variations in water chemistry can be interpreted in terms of water hydrogeology. The application of PCA and HCA to water samples for multivariate association between these factors has been successful. The PCA technique for water samples is shown in Table S1 in the Supporting Information, and the HCA dendrogram is presented in Figure 2. All four components extracted were based on the eigenvalue greater than 1 (significant) and accounted for 69.8% of the total variance. This percentage indicates that the water samples were affected by different factors. Both analyses were performed on a data set using 45 samples, and the following elements were taken as factors: as physiochemical parameters—EC, TDS, and SO_4_^2–^; as PTMs—Mn, B, K, Al, Ca, Mg, Si, Co, Ni, and Zn, to cluster groups of samples with similar characteristics. In HCA, the variables were combined using different methods. The best dendrogram was obtained using the Pearson correlation with the between-group linkage method.

The first cluster group in HCA that was correlated with PC1 had 28.1% total variance, 3.939 of the eigenvalue, and strong positive loadings for EC (0.949), TDS (0.949), Ca (0.905), SO_4_^2–^ (0.651), Mg (0.625), and B (0.529). The second HCA comprised Mn, Al, Si, and Zn, which correlated with PC2, and had high positive loadings of 0.843, 0.772, 0.725, and 0.666, respectively, and a considerable percentage of 20.5% of the total variance in the data set. PC3 explained 10.6% of the total variance and 1.484 of the eigenvalues and gave an inverse relationship between Fe (0.696) and DO (0.776), which is possible in groundwater because Fe dissolves under a smaller amount of oxygen. In this case, this relationship can also be attributed to the fact that the acidity in the water increased and there was Fe dissolution as a result of organic acid formation.^60^ The variables that highly loaded in the fourth cluster and PC4 were Co (0.843) and Ni (0.744), and the percent contribution of PC4 to the total variance was 10.6%. The variables in PC1–PC3 are mostly due to natural occurrences, implying that the pollution source is lithogenic in nature, which is contributed by acidic mining drainage. However, no geologic sources of boron (B), cobalt (Co), and nickel (Ni) elements were found in general in Kirazlı and its surroundings, but Çan basin coals used in the Çan thermal power plant were found to possess the hazardous trace elements cobalt,^65^ boron, and nickel,^66^ which indicates that their presence might be due to anthropogenic pollution.

### 3.3 Assessment of Soil Quality

#### 3.3.1 Background Value (BGV)

The determination of environmental background values (BGVs) is necessary to evaluate PTM pollution in all soils because they represent the PTM concentration in soil, unaltered by human activity (preanthropogenic level).^67^ BGV is significant for geochemical data in distinguishing site-related contamination and giving the baseline concentration for the sampling location. In this study, it was calculated by a normality test (Shapiro–Wilk), which helps understand whether the PTMs show a normal, logarithmically normal, or skewed distribution. Before applying the normality test, outliers were determined by Box–Whisker plots and removed from the raw data. BGV was an arithmetic mean (*M*) if the data were normally distributed, a geometric mean (GM) if the data were distributed logarithmically, or a median if the data showed a skewed distribution. Standard deviation (SD) was changed to the geometric standard deviation (GSD) in a logarithmically distributed data set to define a range. Mean* (M*) and SD* refer to values computed after eliminating the extreme values.^67,68^

The basic statistics and BGVs of the metals are exhibited in Table 3. The results showed that in contrast to Al, Co, Cu, Fe, and Zn, which were normally distributed, Ca, Cd, Mg, Mn, and Ni were logarithmically distributed, and B, Ba, Cr, Pb, and Si showed a skewed distribution. We compared our BGVs with mean values of Chinese soils and Bangkok soils as reference values. Ba (60 mg/kg) has no reference value to compare. The BGVs of Cr (32.50 mg/kg) and Cd (0.30 mg/kg) were found to be slightly greater than values of Bangkok soils. Wang and Wei^69^ measured some of the HMs in Chinese soil and found their concentration as follows: Co, 12.7 mg/kg; Ni, 26.9 mg/kg; and Zn, 74.2 mg/kg. In our study, the Co concentration was 14.2 mg/kg and the Zn concentration was 79.4 mg/kg, while their detected levels were much lower. Wilcke et al.^54^ studied 30 different Bangkok topsoils (0–5 cm) from young deposits of near-pristine materials. The average concentrations of Al and HMs were below our BGVs, except for Ni and Zn (Table 3). However, PTMs in soils can easily dissolve and solutes can transport into the groundwater through porous media with pressure and gravity. Hence, while groundwater is one of the major sources of drinking water, contamination sources close to the groundwater flow affect the potable water quality.^70^

**Table 3:** Statistics of Raw and Background Data (mg/kg)^a^

	statistics of raw data					statistics of background values				
metals	min	max	mean	median	SD	mean* (M*)	SD*	geo. mean (GM)	GSD	Bangkok soils
Al	9370	73 200	30 337.50	29 637.50	17 946.78	**26 440.91**	12 404.61	23 432.46	1.72	13 800
B	4480	39 855	17 377.50	**8530**	14 297.36	17 377.50	14 297.36	12 749.58	2.25	ND
Ba	35.00	4030	419.58	62.50	1138.51	91.36	61.81	76.50	1.82	ND
Ca	810	24 580	4831.67	3015	6497.86	3036.36	1974.66	**2459.22**	2.02	ND
Cd	0.00	1.80	0.43	0.30	0.49	0.33	0.15	**0.30**	1.56	0.29
Co	7.80	20.20	**14.19**	14.40	4.13	14.19	4.13	13.59	1.37	ND
Cr	28.00	147.80	49.47	34.50	33.46	39.22	13.25	37.53	1.62	26.40
Cu	6.40	97.40	41.38	42.90	28.36	**41.38**	28.36	30.20	2.56	41.70
Fe	17 580	63 650	**38 887**	36 653	12 956	38 887	12 956	36 813	1.43	16 100
Mg	130	1637.50	511.88	410	411.00	409.55	218.16	**352.47**	1.82	ND
Mn	70	805	349.17	247.50	263.24	349.17	263.24	**261.83**	2.29	340
Ni	0.60	81.60	14.06	3.10	27.76	2.40	1.53	**1.89**	2.27	24.80
Pb	24.20	74.60	40.63	**32.20**	16.56	40.63	16.56	37.97	1.45	47.80
Si	251 050	496 350	339 939.58	**293 350**	89 810.55	339 939.58	89 810.55	330 024.70	1.28	ND
Zn	13.80	217.20	90.88	79.10	52.23	**79.39**	33.84	69.34	1.87	118

a Bold values represent background values (BGVs).

#### 3.3.2 Assessment of Soil Pollution Indices

Three soil pollution indices (EF, CF, and PLI) were applied to normalize the soil pollution concentration of PTMs (see Table 4). EF is frequently used for management measures of excess metal concentrations in the soil due to man-made effects. In Kirazlı and close to the sampling points, the EF presented various enrichments with respect to the PTMs, but mostly it exhibited minor enrichments. The results with respect to pollution indices for each soil sample are shown in Tables S2–S4. According to Yilgor et al.,^71^ if an EF value is higher than 1.5, it indicates anthropogenic pollution. Ba, Cd, Cr, Cu, and Pb showed mostly anthropogenic pollution with respect to all pollution indices. S2 (collected from the Atıkhisar Dam) has the highest EF for Ba (47.43) and the second highest EF for Ni (6.15); this can be attributed to the high concentrations of Ba and Ni caused by mining tailings via groundwater flows, and its effectiveness decreases on moving toward the dam. Al showed generally minor anthropogenic pollution. The second highest EF was calculated for S1 (taken from the stream connected to the Atıkhisar Dam) as 45.91 for Ni, indicating very severe enrichment, and additionally, EFs for B and Mn were estimated as 4.44 and 3.07, respectively (Table S2). The EF values of PTMs were in good agreement with the previous studies performed in the soil of this region.^41,72^ However, in some sampling points, while soil samples were within the limits of pollution indices (i.e., EF), Al in water samples demonstrated high concentrations. Hence, in the sampling points that showed low EF values in the soil and high Al concentrations in water samples, the pollution source for Al was determined to be lithogenic-based. For instance, S2, S4, S6, S9, and S11 had minor pollution, but in the same points, W32, W12, W14, W42, and W45 had 10.05, 8.12, 2.48, 2.04, and 7.70 mg/L Al concentrations, respectively. These sampling points were located around the mining site. This implies that the mineral containing this metal dissolved and leached away during mine searching processes in the bedrock and passed from the soil to the groundwater by dissolving Al. On the other hand, in the region of samples with both high EF value in soil and high Al concentration in water, it was concluded that the source of pollution was not only lithogenic but also anthropogenic. These samples were S15 (EF_Al_ = 1.8), W18 (11.54 mg/L), and S17 (EF_Al_ = 2.45) – W20 (2.60 mg/L). Regarding the high EF values (>1.5), Mn and Ni enrichment in S1 and S2, B and Ni enrichment in S3 and S8, and Co and Zn enrichment in S8 and S10 because of human-induced activities were deduced. Nevertheless, to confirm that PTMs in sampling locations were contributed by not anthropogenic but geogenic activities, other soil contamination indices, which are *I*_geo_, CF, and PLI, were calculated and discussed.

**Table 4:** Soil Assessment Factors in Terms of PTMs

assessment factors	statistics	Al	B	Ba	Cd	Co	Cr	Cu	Mn	Ni	Pb	Zn
EF	min	0.38	0.71	0.51	0.00	0.34	0.56	0.09	0.23	0.32	0.65	0.18
	max	3.17	4.59	47.43	4.27	1.90	4.85	5.21	6.80	45.91	3.50	3.13
	mean	1.34	2.07	5.58	1.34	1.10	1.71	1.23	1.62	7.63	1.41	1.31
	median	1.08	1.02	1.53	1.33	1.01	1.41	1.02	1.20	2.05	1.08	0.94
	SD	1.01	1.60	13.26	1.13	0.42	1.23	1.35	1.81	15.58	0.82	0.94
*I*_geo_	min	–2.08	–1.51	–1.36	–1.17	–1.45	–0.80	–3.28	–2.02	–2.49	–1.00	–0.81
	max	0.88	1.64	5.48	2.00	–0.08	1.60	0.65	1.63	1.04	0.63	0.17
	mean	–0.62	–0.01	0.24	–0.19	–0.65	–0.17	–1.04	–0.40	–0.58	–0.35	–0.42
	median	–0.42	–0.59	–0.53	–0.17	–0.56	–0.50	–0.53	–0.37	–0.71	–0.59	–0.59
	SD	0.88	1.17	1.85	1.06	0.46	0.70	1.36	1.04	1.19	0.54	0.36
CF	min	0.35	0.53	6.99	0.01	0.55	0.82	0.69	0.37	0.27	0.68	0.86
	max	2.77	4.67	1.77	0.01	1.42	0.72	0.50	4.65	3.07	0.63	1.69
	mean	1.15	2.04	1.04	0.01	1.00	0.58	0.72	1.45	1.33	0.54	1.16
	median	1.12	1.00	18.98	0.01	1.01	0.56	0.47	1.16	0.95	0.28	1.00
	SD	0.68	1.68	3.59	2.08	0.29	1.62	2.56	1.17	1.01	1.45	0.31

Table S3 shows that *I*_geo_ values classified almost all PTMs as unpolluted by human activities. It varied from −2.08 to 0.88 with a mean value of −0.62 for Al, −1.51 to 1.64 with a mean value of −0.01 for B, −1.45 to −0.08 with a mean value of −0.65 for Co, −1.73 to 0.13 with a mean value of −0.66 for Fe, −2.49 to 1.04 with a mean value of −0.58 for Mn, −2.24 to 4.84 with a mean value of 0.48 for Ni, and −3.11 to 0.87 with a mean value of −0.64 for Zn (Table S3). The average values of *I*_geo_ are on the order of Fe < Co < Zn < Al < Mn < B < Ni. It supports the site’s pollution source as either geogenic- or pedogenic-based (*I*_geo_ < 0) mostly, which is similar to the conclusion obtained with EF. In other words, if *I*_geo_ is lower than 0, the pollution is caused by soil weathering.^73^ As seen in Table S3, Ni showed the relatively highest *I*_geo_ values for S1 (*I*_geo_ = 4.84; from strongly to extremely polluted) and S2 (*I*_geo_ = 2.54; from moderately to strongly polluted). Besides Ni, B has the highest artificially deposited pollutant and was recorded in S1, S3, S5, and S6 collected locations.

CF was also considered for assessing the soil pollution by metals in each of the sampling sites with PLI. These indices are commonly used to normalize the metal concentrations. Extreme contamination of Ni in S1 and S2 points was also confirmed by CF and PLI values, which amounted to 43.07 and 8.71, respectively (Table S4). Also, 50% of the points was determined as polluted with respect to PLI, 42% of the points was observed as moderately polluted, while 6% was polluted to a considerable level. Due to PLI, S1–S3, S6, S8, S10, and S12 were found to be polluted. It was contributed by CF, which presented the common metals for contamination, such as Al, Ni, and Zn.

#### 3.3.3 Statistical (Pearson Correlation, PCA, and HCA), XRD, and SEM-EDS Analyses

Pearson correlation, PCA, and HCA analyses were carried out on the soil data set taking into consideration the variables (i.e., metals) Al, Ca, Fe, Mg, Na, Ni, and Si to highlight that these metals were originally in the soil (Figure S1 and Table S5). The Pearson correlation showed positively strong relations between Ni and Ca (0.950), Na and Si (0.752), and Na and Mg (0.63). Since there are just four components with respect to the PCA techniques (Table S5), the Pearson correlation supported the fact that the analyses perfectly matched each other. The PCs accounted for 90.8% of the total variance in the data set. Si, Na, and Mg were clustered as PC1 had 32.5% of the total variance and had strong positive loadings. These analyses also evidence that these metals were naturally found in the soil. For instance, as seen in Figure S2a,b, S1 possesses them as in montmorillonite minerals. XRD analysis showed the minerals, in Table S6, for each soil sample (S1–S12). PC2 also had positively strong loadings between Ni (0.953) and Ca (0.953). PC3 had a considerable proportion of 15.4 of total variance and had strong loading for Fe (0.977), while PC4 represented 13.3% of the total variance for Al (0.934). While both Fe and Al are abundant metals found as minerals in the soil of the region such as montmorillonite, gismondine, nontronite, and kaolinite, they were clustered as independent variables and associated with all metal groups.

Due to these metals being lithogenically present in the soil matrix, the metal pollution was assumed to be caused geogenically, for instance, soil weathering or acidic mining drainage.^74^ However, Ni metal was just found in S1 and S2 naturally, and its values of EF and other pollution assessment factors were found to be very high for Ni, indicating that it was due to both geogenic and anthropogenic factors. Acidic mining drainage forms sulfuric acid when rain or streams come in contact with minerals such as sulfur-rich pyrite, and this acid pollutes the water and soil due to the effect of gravity and forms orange or red precipitates where it passes. While S2 and W32 were from the same stream in Kirazlı, they were close to W33 (around 1 km). According to this, due to the proximity of the samples and the similarity of the features, the Al concentrations of W32 and W33 were due to to acidic mining drainage. Hence, both W32 (10.05 mg/L) and W33 (16.26) had high Al concentrations, which was also contributed by S2 (46,515 mg/kg).

### 3.4 Al Removal from Water Samples with High Aluminum Concentrations

Acidic natural ground or spring waters with high Al concentrations eventually merge into receiving water sources (e.g., a stream entering a lake). Al concentrations will decrease in these water sources due to dilution and precipitation reactions. However, these natural waters with high Al concentration and low pH in the Kirazlı region of Northwestern Turkey are used in rural settlements for the purpose of irrigation of fruits and vegetables, sometimes for domestic purposes, and also as drinking water by animals such as cattle and sheep. In addition, these natural water resources have decreased due to global climate change, so spring waters in these settlements have become important. Therefore, it may be possible to use them when Al is removed from these waters.

Various advanced chemical and physical treatment techniques, such as adsorption with nanomaterials, chemical precipitation, electrolysis, ion exchange, membrane processes, biological methods, and hybrid processes, have been used to eliminate Al from water resources.^75,76^ Especially, adsorption has emerged as the most efficient technique due to its simplicity of application, environmentally friendly nature, and low cost. Various adsorbents, such as granular activated carbon,^5^ magnetic iron oxide nanoparticles,^77^ iron-modified carbons,^78^ natural zeolite,^79^ and carbon nanotubes,^80^ have been utilized for Al removal in waters. However, these adsorbents have the disadvantages of relatively low adsorption capacity, regeneration difficulties, disposal of precipitated wastes, and requirement of a long operation time, hence limiting their real-scale practical applications.^79^ Al removal techniques like sedimentation, electrolysis, membrane processes, and filtering are also moderately effective, complex, and expensive.^81^ On the other hand, precipitation with pH adjustment and ion exchange are promising methods for Al removal considering their ease of application, relatively low costs, and considerably high removal efficiencies.^5^ Namely, by adjusting the pH to an alkaline level with the help of calcium carbonate and lime, Al from acidic effluents can often be removed from the water by forming an insoluble precipitate.^82^ Furthermore, the removal of heavy metals in waters using an ion-exchange process with different synthetic or natural resins is one of the most promising methods owing to its features of easily recoverable end-products, the possibility of reuse after the regeneration step, and effectiveness. Heavy metal removal by various ion-exchange resins has been examined by many previous studies.^83,84^ However, there is no available research on the removal of Al from real water resources.^85,86^ Moreover, the Al removal performance of the ion-exchange process in the presence of various metals has not been investigated adequately. To the best of our knowledge, the use of ion-exchange resins for Al removal in real wastewater or waters is also very limited. There are only two studies in the literature discussing Al removal by the ion-exchange process from water resources.^87,88^ Use of the ion-exchange process for Al-containing real water treatment is challenging due to the high variability of electrical conductivity, pH, and competition between ions. Overall, the originality of this paper lies in investigating the selective removal of Al by the Amberlite IR-120H resin and pH adjustment from real groundwater resources in the presence of other ions.

**Figure 3:**
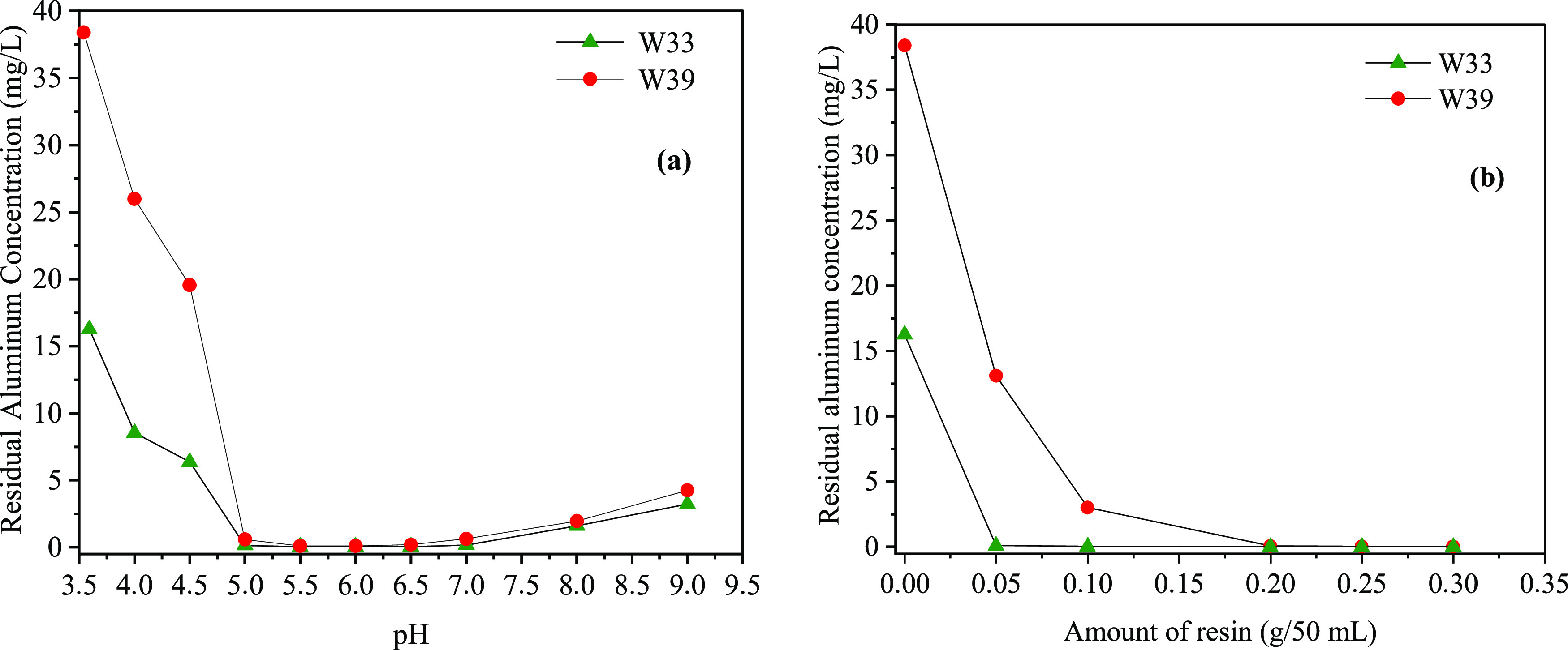
Studies of Al removal from water samples: (a) precipitation with pH adjustment, and (b) ion-exchange resin (Amberlite IR-120H).

According to the results of the Al pollution detection study carried out in the study area, the removal results were evaluated by applying precipitation with pH adjustment and treatment with the ion-exchange process on the W33 and W39 samples with the highest Al concentration. The results of Al removal by adjusting the pH are shown in Figure 3a, and the removal results with the ion-exchange resin are shown in Figure 3b. The pH value of the spring water (W33) is 3.59, and the total dissolved Al concentration is 16.26 mg/L. It can be seen from Figure 3a that between pH 5.5 and 7.0, the Al concentration in water drops below 0.10 mg/L, and the Al concentration in water above pH 7 increases. The pH and Al concentrations of the other spring water sample (W39) are 3.54 and 38.38 mg/L, respectively. With pH adjustment, the dissolved Al concentration in water between pH 5.5 and 6 is 0.11 mg/L, and it is above 0.50 mg/L at pH 5. The Al concentration of the W39 water sample is higher than that of the other water sample. In this case, it is possible to reduce up to a certain Al concentration only by adjusting the pH values of the water samples. The acidic and basic pH values increase the soluble forms of Al and prevent its precipitation as Al(OH)_3(s)_. At pH values below 3.5, the Al ion is the predominant species. Monomeric and polymeric Al species such as Al*_n_*(OH)*_n_*^(3–*n*)+^: Al(OH)_2_^+^, Al(OH)_2_^+^, Al(OH)_4_^–^, Al_6_(OH)_15_^3+^, Al_7_(OH)_17_^4+^, Al_8_(OH)_20_^4+^, Al_13_(OH)_34_^5+^, and Al(OH)_3(s)_ are formed in solution between pH 3.5 and 14.^89^ The solubility product of Al hydroxide, Al(OH)_3(s)_, is *K*_sp_ 1.9 × 10^–33^ at 25 °C. The pH of minimum solubility of the solid Al(OH)_3(s)_ is about 5.0–6.5, and the total soluble Al^3+^ concentration is (3 × 10^–6^) to (3 × 10^–4^) M (or 0.025–2.5 mg/L) between pH 5 and 9.

The results obtained from W33 (pH 3.59) and W39 (pH 3.54) for Al removal using different ion-exchange resin amounts are shown in Figure 3b. For W39, at a dosage of 0.1 g of resin, the Al concentration is reduced below 0.10 mg/L, while for W33, an Al effluent concentration of 0.10 mg/L is achieved at 0.05 g of resin dosage. Al ion exchanger capacities (mg/g at 0.05, 0.10, 0.20, 0.25, and 0.30 g/50 mL) and resin amounts were calculated as 13.76, 11.93, 6.70, 5.37, and 4.48 mg/g for the W39 spring water sample (16.26 mg Al/L) and 11.98, 6.02, 3.02, 2.42, and 2.01 mg/g for the W33 spring water sample (38.38 mg Al/L), respectively. Considering the results obtained above, it is seen that Al removal by precipitation is not enough, especially at high Al concentrations. In this case, it would be more appropriate to use the precipitation at pH 5.0–7.0 and then the ion-exchange process for the reliable use of water after Al precipitation by pH adjustment. Thus, the usage time of the resin will increase.

Overall, these results clearly showed that pH adjustment only did not work in the case of high Al contamination and relatively low removal was achieved when compared with the ion-exchange-resin Al removal performance. In addition, making a continuous pH adjustment to increase the Al removal efficiency will also increase the operating cost considerably. Similarly, when Al removal is performed using only an ion-exchange method, although high Al removal efficiencies are achieved, the resin lifetime ends in a short time, creating a serious problem in real applications. Therefore, these results revealed that the environmentally most compatible and cost-effective solutions include a combination of pH adjustment and ion-exchange process. Furthermore, although the scale of the processes in some of these situations is comparatively small, as in the case of rare-earth components or noble metals, the utility of the recovered metals is extremely great. The ion-exchange process is especially appropriate for separation of metal ions with a low handling and high value. Ion-exchange procedures are widely used in hydrometallurgy, and their use is increasing yearly.

## 4 Conclusions

This study offers a multivariate simultaneous statistical evaluation of PTMs and physicochemical parameters using PCA and HCA classification to assess the water and soil quality of Kirazlı and the villages of Çanakkale. The impacts of geogenic and anthropogenic sources on various parameters were investigated in short-term observation monitoring data. Promising basic treatment processes were experimented to remove Al pollution in highly polluted waters. Hence, simple traditional methods can be implemented at lower costs in villages where there is mining activity, affording safe water supply for animals or agricultural irrigation.

## Supporting Information Available

Soil matrix XRD and SEM-EDS results; environmental risk associated with enrichment factor, geoaccumulation index, contaminant factor, and pollution load index; and principal component analysis and hierarchical cluster analysis technique for water and soil samples (PDF)
