# Supplementary material for: Identifying Geogenic and Anthropogenic Aluminum Pollution on Different Spatial Distributions and Removal of Natural Waters and Soil in Çanakkale, Turkey
Source: ACS Omega. 2023 Feb 22;8(9):8557–68. doi: 10.1021/acsomega.2c07707 (PMC9996766; doi:10.1021/acsomega.2c07707)
Supplement: Supplementary file 1 — ao2c07707_si_001.pdf [file ao2c07707_si_001.pdf]

Supplementary Material

for

Identifying Geogenic and Anthropogenic Aluminum Pollution on Different Spatial Distribution and Removal of Natural Waters and Soil in Çanakkale, Turkey

Sezin Hızlı[a], Aybike Gül Karaoğlu[a], Ayşegül Yağmur Gören[b,*], Mehmet Kobya[a,c,*]

[a]Gebze Technical University, Department of Environmental Engineering, 41400, Gebze-Turkey
[b]Izmir Institute of Technology, Department of Environmental Enginnering, Urla, İzmir, Turkey
[c]Kyrgyz-Turkish Manas University, Department of Environmental Engineering, Bishkek, Kyrgyzstan

*Corresponding author:

kobya@gtu.edu.tr, Gebze Technical University, Department of Environmental Engineering, 41400, Kocaeli, Turkey

yagmurgoren@iyte.edu.tr, İzmir Institute of Technology, Department of Environmental Engineering, Urla, İzmir, Turkey

Table S1. PCA technique for water.

Rotated Component Matrixa				
Variable	Component			
	PC1	PC2	PC3	PC4
EC	**0.949**	0.144	0.083	0.032
TDS	**0.949**	0.144	0.076	0.032
Ca	**0.905**	-0.028	0.036	-0.072
SO4	**0.651**	**0.55**	0.076	0.137
Mg	**0.625**	-0.116	-0.188	-0.375
B	**0.529**	-0.166	0.457	0.105
Mn	0.208	**0.843**	0.157	-0.048
Al	-0.121	**0.772**	-0.209	-0.037
Si	0.109	**0.725**	-0.02	0.126
Zn	-0.024	**0.666**	0.074	-0.063
DO	-0.231	0.139	**-0.776**	0.129
Fe	-0.047	0.386	**0.696**	0.034
Co	0.165	-0.143	-0.217	**0.843**
Ni	-0.266	0.139	0.112	**0.744**
Total	3.939	2.87	1.484	1.482
% of Variance	28.137	20.498	10.604	10.584
Cumulative %	28.137	48.635	59.238	69.822
Extraction Method: Principal Component Analysis.				
Rotation Method: Quartimax with Kaiser Normalization.				

Bold values are strong correlations (r >0.5).

Table S2. Enrichment Factors (EF > 1.5 was highlighted with red color).

Soil No	Al	B	Ba	Cd	Co	Cr	Cu	Mn	Ni	Pb	Zn
S1	0.38	4.44	2.66	1.43	1.08	4.85	1.29	3.07	45.91	1.01	0.66
S2	1.24	0.77	47.43	4.27	1.00	1.37	1.37	1.86	6.15	1.03	0.80
S3	0.51	3.63	0.78	2.09	0.79	0.75	0.45	1.23	1.81	1.80	1.31
S4	0.52	0.88	1.93	1.23	0.34	0.56	0.09	0.40	ND	0.84	0.46
S5	0.42	4.05	0.83	0.00	0.96	0.94	0.17	0.29	0.32	0.88	1.00
S6	0.58	4.59	0.90	0.73	1.27	0.94	1.50	1.29	1.38	1.14	2.97
S7	1.80	1.06	1.83	1.73	0.73	1.44	1.37	0.89	ND	1.23	0.88
S8	2.92	1.95	2.03	1.49	1.62	2.22	5.21	6.80	2.80	3.50	3.13
S9	0.99	0.71	0.51	0.58	1.03	0.87	0.97	0.23	0.37	0.65	0.82
S10	2.45	0.87	5.82	1.12	1.90	3.16	0.32	1.17	ND	1.58	1.65
S11	1.16	0.97	0.97	1.42	1.50	1.49	1.07	1.41	ND	0.84	0.18
S12	3.17	0.96	1.24	0.00	0.95	1.89	0.95	0.81	2.30	2.39	1.83
Min	0.38	0.71	0.51	0.00	0.34	0.56	0.09	0.23	0.32	0.65	0.18
Max	3.17	4.59	47.43	4.27	1.90	4.85	5.21	6.80	45.91	3.50	3.13
Mean	1.34	2.07	5.58	1.34	1.10	1.71	1.23	1.62	7.63	1.41	1.31
Median	1.08	1.02	1.53	1.33	1.01	1.41	1.02	1.20	2.05	1.08	0.94
SD	1.01	1.60	13.26	1.13	0.42	1.23	1.35	1.81	15.58	0.82	0.94

Table S3. Geoaccumulation Index [I_{geo} values were highlighted with yellow colour (0-1), with blue colour (1-2), with green colour (2-3), and with red colour (4-5)].

Soil No	Al	B	Ba	Cd	Co	Cr	Cu	Fe	Mn	Ni	Pb	Zn
S1	-2.08	1.47	0.74	-0.17	-0.56	1.60	-0.31	-0.68	0.94	4.84	-0.67	-1.27
S2	0.23	-0.47	5.48	2.00	-0.08	0.37	0.37	-0.08	0.81	2.54	-0.04	-0.40
S3	-1.19	1.64	-0.58	0.83	-0.56	-0.63	-1.37	-0.22	0.08	0.63	0.63	0.16
S4	-0.83	-0.06	1.08	0.42	-1.45	-0.71	-3.28	0.13	-1.21	ND	-0.13	-0.99
S5	-1.83	1.43	-0.85	ND	-0.65	-0.67	-3.11	-0.58	-2.39	-2.24	-0.77	-0.58
S6	-1.49	1.50	-0.85	-1.17	-0.36	-0.80	-0.12	-0.70	-0.34	-0.24	-0.51	0.87
S7	-0.10	-0.86	-0.08	-0.17	-1.41	-0.42	-0.50	-0.95	-1.13	ND	-0.66	-1.13
S8	-0.19	-0.77	-0.71	-1.17	-1.03	-0.58	0.65	-1.73	1.04	-0.24	0.08	-0.08
S9	-0.40	-0.88	-1.36	-1.17	-0.34	-0.59	-0.43	-0.38	-2.49	-1.83	-1.00	-0.67
S10	-0.03	-1.51	1.22	-1.17	-0.39	0.34	-2.96	-1.32	-1.09	ND	-0.66	-0.60
S11	-0.44	-0.71	-0.71	-0.17	-0.08	-0.08	-0.57	-0.66	-0.17	ND	-0.92	-3.11
S12	0.88	-0.84	-0.47	ND	-0.85	0.14	-0.85	-0.78	-1.09	0.42	0.48	0.09
Min	-2.08	-1.51	-1.36	-1.17	-1.45	-0.80	-3.28	-1.73	-2.49	-2.24	-1.00	-3.11
Max	0.88	1.64	5.48	2.00	-0.08	1.60	0.65	0.13	1.04	4.84	0.63	0.87
Mean	-0.62	-0.01	0.24	-0.19	-0.65	-0.17	-1.04	-0.66	-0.58	0.48	-0.35	-0.64
Median	-0.42	-0.59	-0.53	-0.17	-0.56	-0.50	-0.53	-0.67	-0.71	0.09	-0.59	-0.59
SD	0.88	1.17	1.85	1.06	0.46	0.70	1.36	0.51	1.19	2.30	0.54	0.99

Table S4. Contaminant Factor (CF) and Pollution Load Index (PLI) (highly polluted& very high painted with red, considerable polluted painted with orange and moderate polluted painted with yellow).

**Soil No**	Al	B	Ba	Cd	Co	Cr	Cu	Fe	Mn	Ni	Pb	Zn	PLI
S1	0.35	4.16	2.50	0.01	1.01	2.46	0.84	0.94	2.88	43.07	0.51	0.62	1.30
S2	1.76	1.08	67.17	0.03	1.42	1.05	1.34	1.42	2.64	8.71	0.78	1.13	1.71
S3	0.66	4.67	1.00	0.01	1.01	0.52	0.40	1.29	1.58	2.32	1.24	1.68	0.76
S4	0.84	1.44	3.17	0.01	0.55	0.50	0.11	1.64	0.65	ND	0.74	0.75	0.62
S5	0.42	4.06	0.83	ND	0.96	0.51	0.12	1.00	0.29	0.32	0.47	1.00	0.62
S6	0.53	4.23	0.83	0.00	1.17	0.47	0.95	0.92	1.18	1.27	0.56	2.74	0.75
S7	1.40	0.83	1.42	0.01	0.56	0.61	0.73	0.78	0.69	ND	0.51	0.69	0.57
S8	1.32	0.88	0.92	0.00	0.73	0.54	1.62	0.45	3.07	1.27	0.85	1.42	0.77
S9	1.14	0.82	0.58	0.00	1.18	0.54	0.77	1.15	0.27	0.42	0.40	0.94	0.54
S10	1.47	0.53	3.50	0.00	1.14	1.03	0.13	0.60	0.71	ND	0.51	0.99	0.67
S11	1.10	0.92	0.92	0.01	1.42	0.77	0.70	0.95	1.34	ND	0.43	0.17	0.60
S12	2.77	0.84	1.08	ND	0.83	0.89	0.57	0.87	0.71	2.01	1.12	1.60	1.00
Min	0.35	0.53	6.99	0.01	0.55	0.82	0.69	0.45	0.27	0.32	0.68	0.17	
Max	2.77	4.67	1.77	0.01	1.42	0.72	0.50	1.64	3.07	43.07	0.63	2.74	
Mean	1.15	2.04	1.04	0.01	1.00	0.58	0.72	1.00	1.33	7.42	0.54	1.14	
Median	1.12	1.00	18.98	0.01	1.01	0.56	0.47	0.94	0.95	1.64	0.28	1.00	
SD	0.68	1.68	3.59	-	0.29	1.62	2.56	0.33	1.01	14.65	1.45	0.66	

Table S5. PCA technique for soil matrix.

Rotated Component Matrixa				
Variable	Component			
	PC1	PC2	PC3	PC4
Na	**0.904**	0.115	-0.011	0.172
Mg	**0.844**	0.032	0.198	-0.009
Si	**0.785**	0.388	-0.254	0.015
Ni	0.182	**0.962**	0.056	-0.092
Ca	0.218	**0.953**	-0.083	-0.121
Fe	0.027	-0.033	**0.977**	-0.092
Al	0.212	-0.255	-0.113	**0.934**
Total	2.274	2.065	1.08	**0.934**
% of Variance	32.483	29.503	15.431	13.337
Cumulative %	32.483	61.986	77.417	90.754
Extraction Method: Principal Component Analysis.				
Rotation Method: Quartimax with Kaiser Normalization.				

Bold values are strong correlations (r >0.5).

Table S6. XRD results of soil samples.

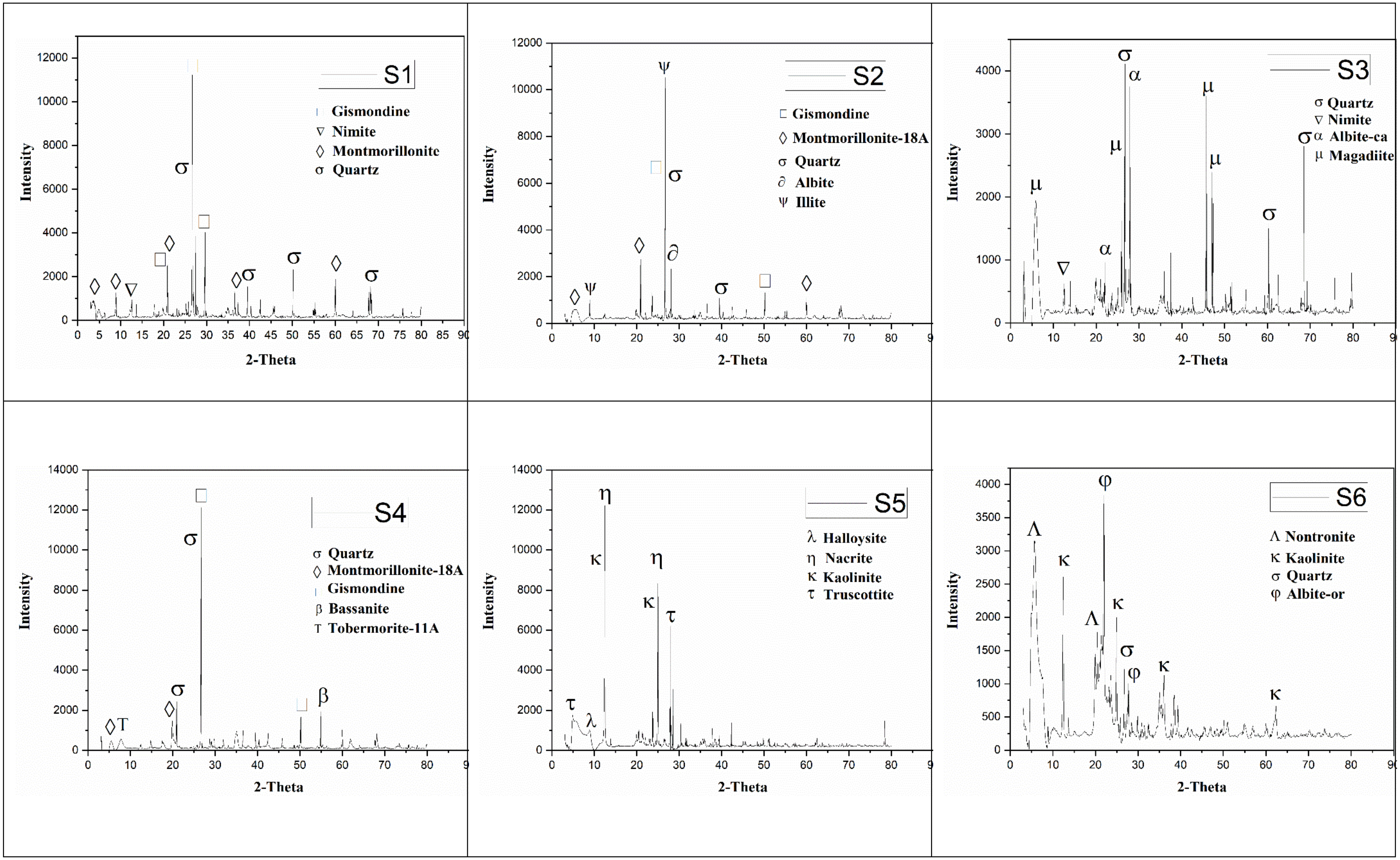

Table S6. XRD results of soil samples (continued).

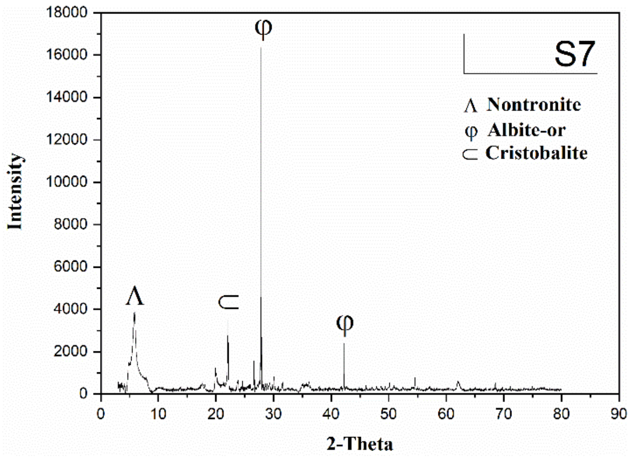

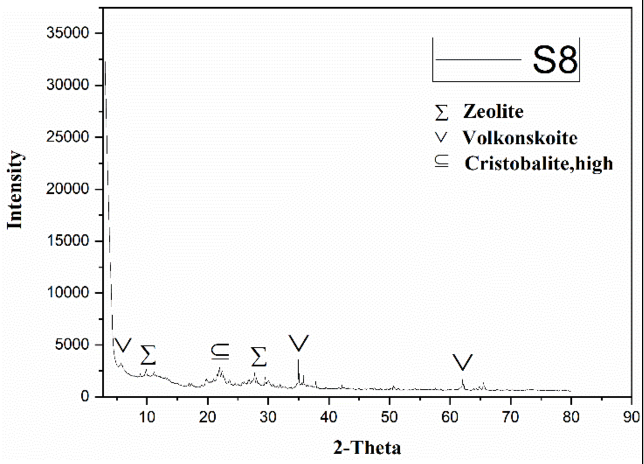

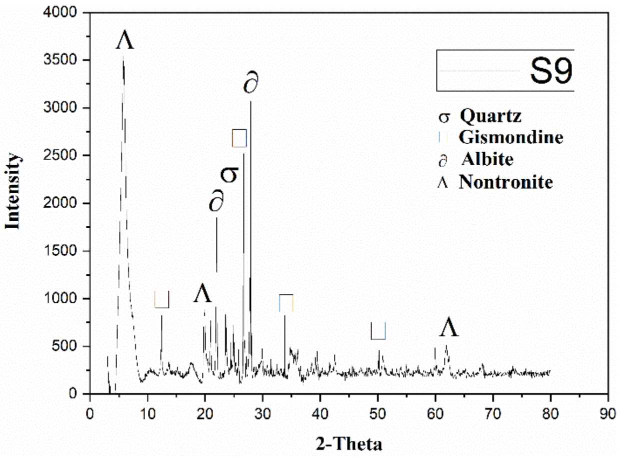

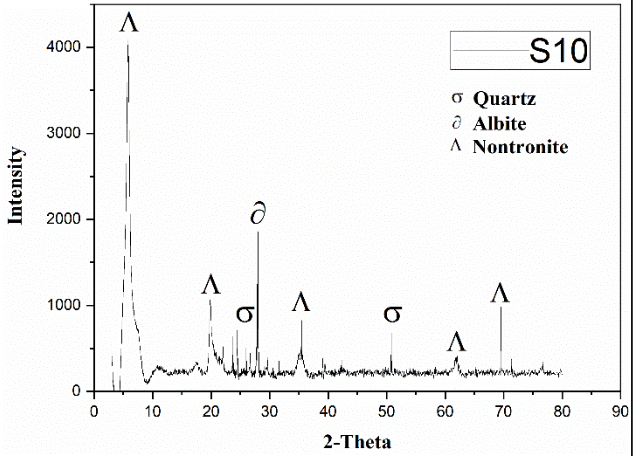

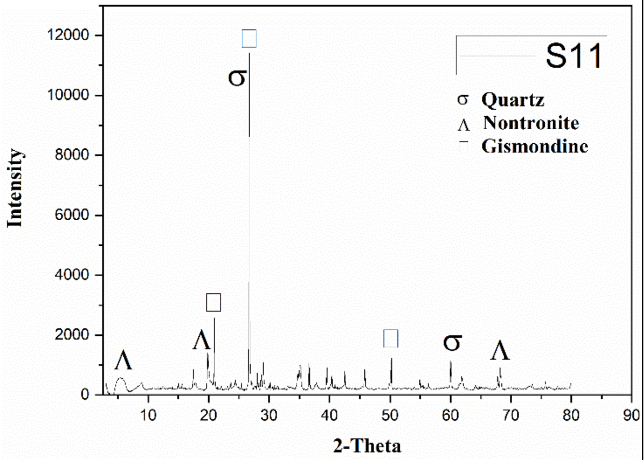

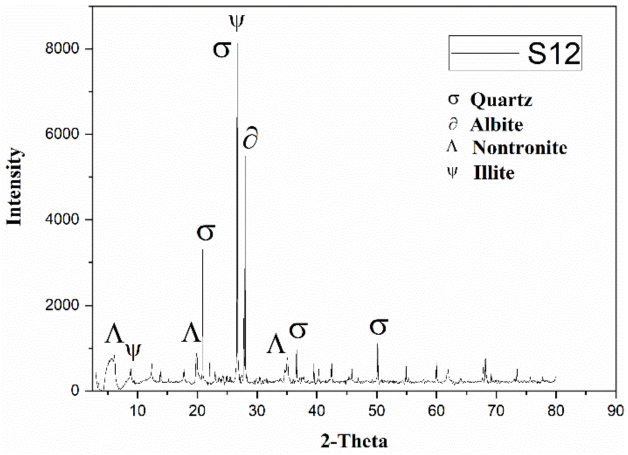

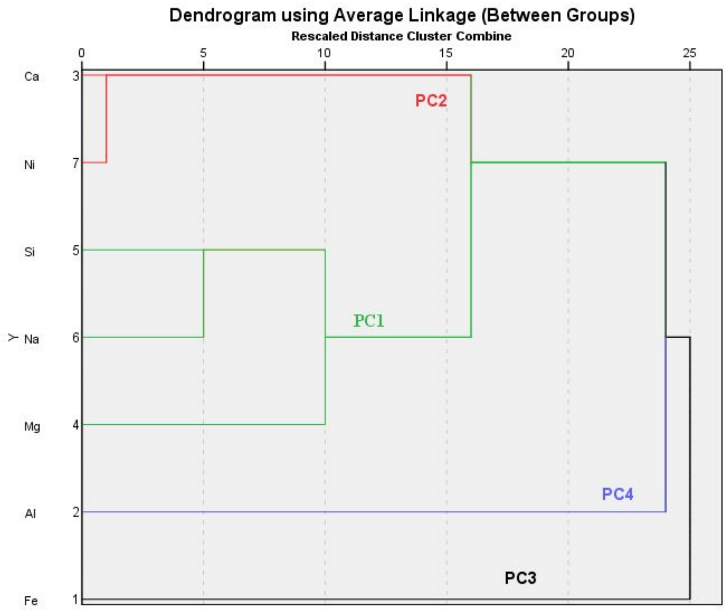

Figure S1. HCA technique for metal determination in soils.

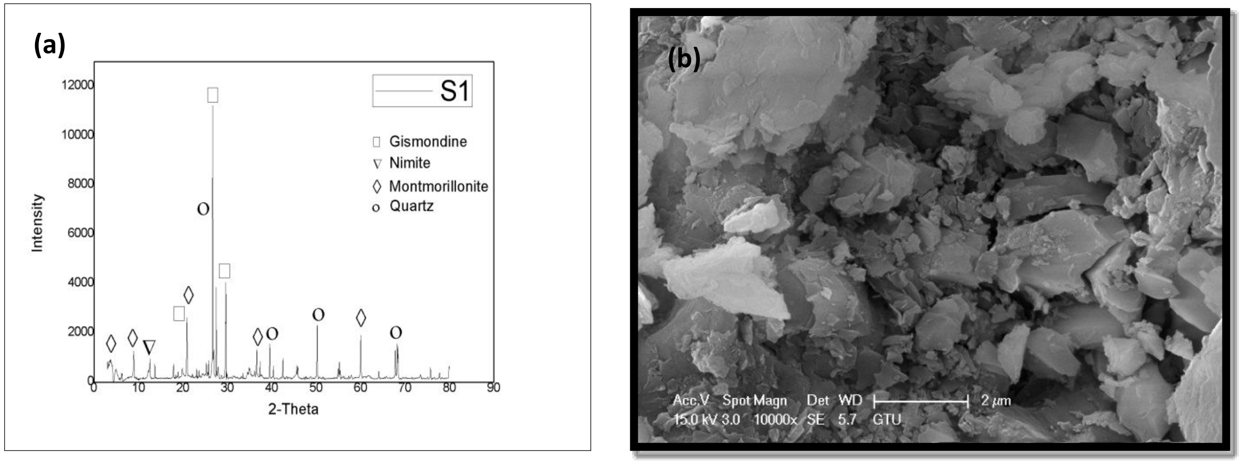

Figure S2. (a) XRD and (b) SEM-EDS of S1.
